# Wide Variations in Compliance with Tuberculosis Screening Guidelines and Tuberculosis Incidence between Antiretroviral Therapy Facilities — Côte d’Ivoire

**DOI:** 10.1371/journal.pone.0157059

**Published:** 2016-06-08

**Authors:** Andrew F. Auld, Michela Blain, Kunomboa Alexandre Ekra, Joseph Sylvain Kouakou, Virginie Ettiègne-Traoré, Moise Zanga Tuho, Fayama Mohamed, Ray W. Shiraishi, Jennifer Sabatier, Joseph Essombo, Georgette Adjorlolo-Johnson, Richard Marlink, Tedd V. Ellerbrock

**Affiliations:** 1 Division of Global HIV & Tuberculosis, Center for Global Health, Centers for Disease Control and Prevention, Atlanta, Georgia, United States of America; 2 Division of Global HIV & Tuberculosis, Center for Global Health, Centers for Disease Control and Prevention, Abidjan, Côte d’Ivoire; 3 Elizabeth Glaser Pediatric AIDS Foundation, Abidjan, Côte d’Ivoire; 4 Ministry of Health, National Program for Medical Care of Persons Living with HIV/AIDS, Abidjan, Côte d’Ivoire; 5 Directorate General of Budget and Finance, Department of Economy and Finance, Abidjan, Côte d’Ivoire; 6 Elizabeth Glaser Pediatric AIDS Foundation, Los Angeles, California, United States of America; Johns Hopkins Bloomberg School of Public Health, UNITED STATES

## Abstract

**Background:**

In Côte d’Ivoire, tuberculosis (TB) is a common cause of death among HIV-infected antiretroviral therapy (ART) enrollees. Ivorian guidelines recommend screening for TB and initiation of TB treatment before ART initiation. Compliance with these guidelines can help reduce TB-related mortality during ART and possibly nosocomial TB transmission.

**Methods and Findings:**

In a retrospective cohort study among 3,682 randomly selected adults (≥15 years old) starting ART during 2004–2007 at 34 randomly selected facilities, documentation of TB screening completion, prevalence of active TB at ART initiation, and incidence of TB during ART were evaluated. At ART initiation, median age was 36 years, 67% were female, and median CD4 count was 135 cells/μL. Among all 3,682 enrollees, 73 (2%) were on TB treatment at the time of referral to the ART facility. Among the 3,609 not on TB treatment, 1,263 (36%) were documented to receive some TB screening before ART initiation; 21% were screened for cough, 21% for weight loss, 18% for fever, 18% for TB contacts, and 12% for night sweats. Among the 1,263 screened, 111 (11%) were diagnosed with TB and started TB treatment before ART. No associations between patient characteristics and probability of being screened were noted. However, documentation of TB screening completion before ART varied widely by ART facility from 0–100%. TB incidence during ART was 3.0 per 100 person-years but varied widely by ART facility from 0/100 person-year to 13.1/100 person-years.

**Conclusions:**

Screening for TB before ART initiation was poorly documented. Facility-level variations in TB screening documentation suggest facility-level factors, such as investment in training programs, might determine documentation practices. Targeting under-performing ART facilities with improvement activities is needed. Variations among facilities in TB incidence warrant further research. These incidence variations could reflect differences between facilities in TB screening, diagnostic tests, documentation practices, or TB risk possibly related to infection control practices or local community TB incidence.

## Introduction

Côte d’Ivoire is a country of 22.8 million people, with 460,000 people living with HIV (PLHIV), an adult HIV prevalence of 3.5%, and about 28,000 HIV-related deaths per year [1,2]. In response to the HIV pandemic, the Ivorian Ministry of Health (MOH) and partners have attempted to rapidly scale-up access to antiretroviral therapy (ART), and despite significant challenges, including two recent civil wars, had enrolled about 140,710 persons on ART by 2014, including 107,453 adults, which corresponds to an ART coverage among all adults with HIV of about 36% [1,2]. Despite significant progress in scaling up ART, the HIV epidemic continues to fuel the pre-existing tuberculosis (TB) epidemic in Côte d’Ivoire, which in 2014 had an annual case notification rate of 165 per 100,000 people [3]. In sub-Saharan Africa, TB remains the most common cause of death among HIV-infected persons, including those who are receiving antiretroviral therapy (ART) [4–6].

In line with World Health Organization (WHO) guidelines, the Ivorian Ministry of Health (MOH) recommends routine TB symptom screening for all PLHIV at every clinical visit, and initiation of TB treatment before ART initiation for those diagnosed as having TB. Compliance with these guidelines can reduce morbidity and mortality from TB during ART [6,7]. Therefore, evaluating compliance with TB screening guidelines is an important program evaluation activity and can indicate opportunities for program improvement [8].

In addition, although the Ivorian MOH does not yet recommend isoniazid preventive therapy (IPT), due to concerns that IPT might select for resistant bacilli when given to patients with undiagnosed active TB, the results from the recent TEMPRANO trial might spur reconsideration of IPT guidelines [9]. The TEMPRANO trial reported a 35% reduction in severe HIV morbidity when eligible HIV-infected patients were prescribed IPT [9]. Since TB screening is necessary before IPT prescription, an evaluation of baseline compliance with TB screening procedures is important if the country were to plan IPT scale-up activities.

In addition to evaluating compliance with TB screening guidelines, evaluating the prevalence and incidence of TB during ART is important for understanding TB morbidity among ART enrollees [10]. Previous studies in Côte d’Ivoire evaluating TB burden and predictors among ART enrollees have been sub-national and have not examined site-level variations in TB incidence during ART [11,12]. Understanding variations in TB incidence by facility might reveal geographic variations in community TB transmission or differences between facilities in nosocomial TB transmission.

Therefore, we analyzed data from the first nationally representative retrospective cohort study of adult ART enrollees in Côte d’Ivoire [13] to assess clinician compliance with TB symptom screening guidelines, the prevalence and determinants of active TB among new adult ART enrollees, incidence of TB during ART, and patient- and site-level determinants of incident TB.

## Methods

### Ethics Approval

This study was approved by the Institutional Review Board (IRB) of the United States Centers for Disease Control and Prevention (CDC) (protocol #5512), the Harvard School of Public Health IRB, and the Ivorian Ethics Review Committee (*Comité National d’Éthique des Sciences de la Vie et de la Santé*). Patient informed consent was not required as only routine, anonymized, monitoring data were collected and analyzed.

### Eligibility for ART

During 2004–2007, patients diagnosed with World Health Organization (WHO) stage IV HIV disease, stage III disease with CD4^+^ T-cell (CD4) counts <350/μL, or stage I or II disease with CD4 counts <200/μL, were eligible for ART [7]. Prescription of co-trimoxazole (CTX) was indicated for all ART patients with CD4 counts ≤350/μL.

### Patient Monitoring

At ART initiation, monthly for the first 3 months, and quarterly thereafter, standardized MOH-recommended medical records were completed to monitor disease progression or improvement. Patients collected medications monthly from clinic pharmacies where the date of scheduled antiretroviral (ARV) pick-up appointments and actual ARV pick-up dates were documented.

Clinicians were recommended to document whether TB screening was performed at each clinic visit and which TB diagnostic tests were performed. Recommended TB screening practices for adults included assessment for (1) cough, (2) loss of weight, (3) fever, (4) TB contact history, and (5) night sweats. Recommended TB tests for all adults screening positive for any of the TB symptoms (i.e., presumptive TB patients) included: (1) two sputum samples sent for smear microscopy, (2) one sputum sent for TB culture, and (3) chest X-ray for patients who were smear-negative. If extra-pulmonary TB was suspected, lymph node aspiration, lumbar puncture and abdominal ultrasound were additional diagnostic tests available at some facilities. In reality, only two laboratories provided TB culture; very few presumptive TB patients would therefore have had sputum samples sent for TB culture. Per Ivorian guidelines, if TB symptom screening was not documented in the medical record, it was considered not done for both patient management and program monitoring purposes.

### Study Design and Population

This was a retrospective cohort study. Patient-level data were abstracted from standardized, MOH-recommended medical records onto study questionnaires by trained abstractors from November 2009 through March 2010. Only medical records of adult patients, ≥ 15 years old at ART initiation, who started ART during 2004–2007, were eligible.

### Sample Size

Sample size calculations were performed using Epi InfoTM software (CDC, Epi Info 2008, Version 3.5.1, Atlanta, GA). Evaluation of TB incidence was a secondary aim of this retrospective cohort study; the primary study question was to estimate 12-month ART attrition (death or loss to follow-up). To achieve a 95% confidence interval (CI) of ±3% around the estimate for 12-month attrition, assuming a design effect of 1.5 [8], and a conservative (i.e., higher than expected) 12-month attrition percentage of 50% [9], a sample size of ≥ 2,301 patient records was needed. Available funding allowed a sample size of 4,000 medical records.

### Sampling

Of 124 ART facilities in the country by December 31, 2007, 78 had provided ART to ≥50 adults. Only 833 (2.3%) of all 36,943 adult patients who had received ART by December 31, 2007, were enrolled at facilities that had supported <50 patients on ART by this time. To maintain feasibility, 35 (45%) of the 78 eligible facilities were selected, using a two-stage sampling strategy. In stage one, the 78 eligible facilities were divided into three strata based on which type of organization was largely responsible for implementing the ART program at the site (non-governmental organization, Ministry of Health (MOH), or the Global Fund for AIDS, TB, and Malaria (GFATM)). Within these three strata, sub-strata were created according to site size (number of ART patients ever enrolled). Site size sub-strata were: small (50–250), medium (251–1,000), and large (>1,000). Within each sub-stratum, SAS software version 9.2 (SAS Institute Inc., Cary, NC) was used to select facilities using probably-proportional-to-size sampling. Of the selected 35 facilities, 34 agreed to participate.

In stage two, simple random sampling was used to select 4,000 medical records from the 34 selected facilities. Clinic-level sample frames of eligible ART patients were derived from paper or electronic ART patient registers located at the clinic. The total number of medical records selected in each sub-stratum was proportional to the number of eligible records in the corresponding sub-stratum in the general adult ART population by 2007.

### Treatment Outcome Measures

Any patient taking TB treatment for pulmonary or extrapulmonary TB at ART initiation was considered to have prevalent TB at ART start. After ART initiation, the first occurrence of TB treatment for pulmonary or extrapulmonary TB was considered incident TB.

### Exposure Variables

Variables routinely collected on the MOH-recommended ART records, including age at ART start, sex, previous TB diagnoses, weight, World Health Organization (WHO) stage, CD4 count, hemoglobin, and (CTX) prescription, were assessed as possible risk factors for prevalent and incident TB. Site level variables including site size (number of adult ART patients ever enrolled), presence of stock outs in the last 12 months, and patient to health care worker ratio were also assessed as possible predictors of incident TB.

### Analytic Methods

Data were analyzed using STATA software version 11.0 (StataCorp, 2009, Stata Statistical Software, Release 11, College Station, TX). The anonymized dataset is available upon request from the analysis working group, comprising the corresponding author, MOH representatives, and representatives from CDC.

After examining patterns and predictors of missing baseline covariate data, missing data, which are reported for each baseline covariate of interest in Table 1, were assumed to be missing at random (MAR), and were imputed using multiple imputation with chained equations [14]. The *ice* [15] procedure in Stata was used to create 20 imputed datasets. The imputation model included incident TB as the event indicator, all study variables, and the Nelson-Aalen estimate of cumulative hazard [16].

Controlling for survey design, associations between baseline covariates and active TB at ART start (prevalent TB) were assessed using logistic regression. Survey-adjusted logistic regression was also used to assess associations between baseline covariates and being successfully screened for TB. Controlling for survey design, Cox proportional hazards regression models were used to estimate adjusted hazard ratios and 95% confidence intervals (CI). The proportional hazards assumption was assessed using visual methods and the Grambsch and Therneau test [17]. Estimates were combined across the imputed datasets according to Rubin’s rules using the mim procedure in STATA [18].

## Results

Data for 3,682 adults enrolled in ART during 2004–2007 were abstracted and analyzed. At ART initiation, median age was 36 (interquartile range: 31‒43), 67% of enrollees were female, 5% were HIV-2-infected or dually HIV-1&2 reactive, and the median CD4 count was 137/μL (IQR 56‒234).

### Documentation of TB Symptom Screening

**Fig 1:**
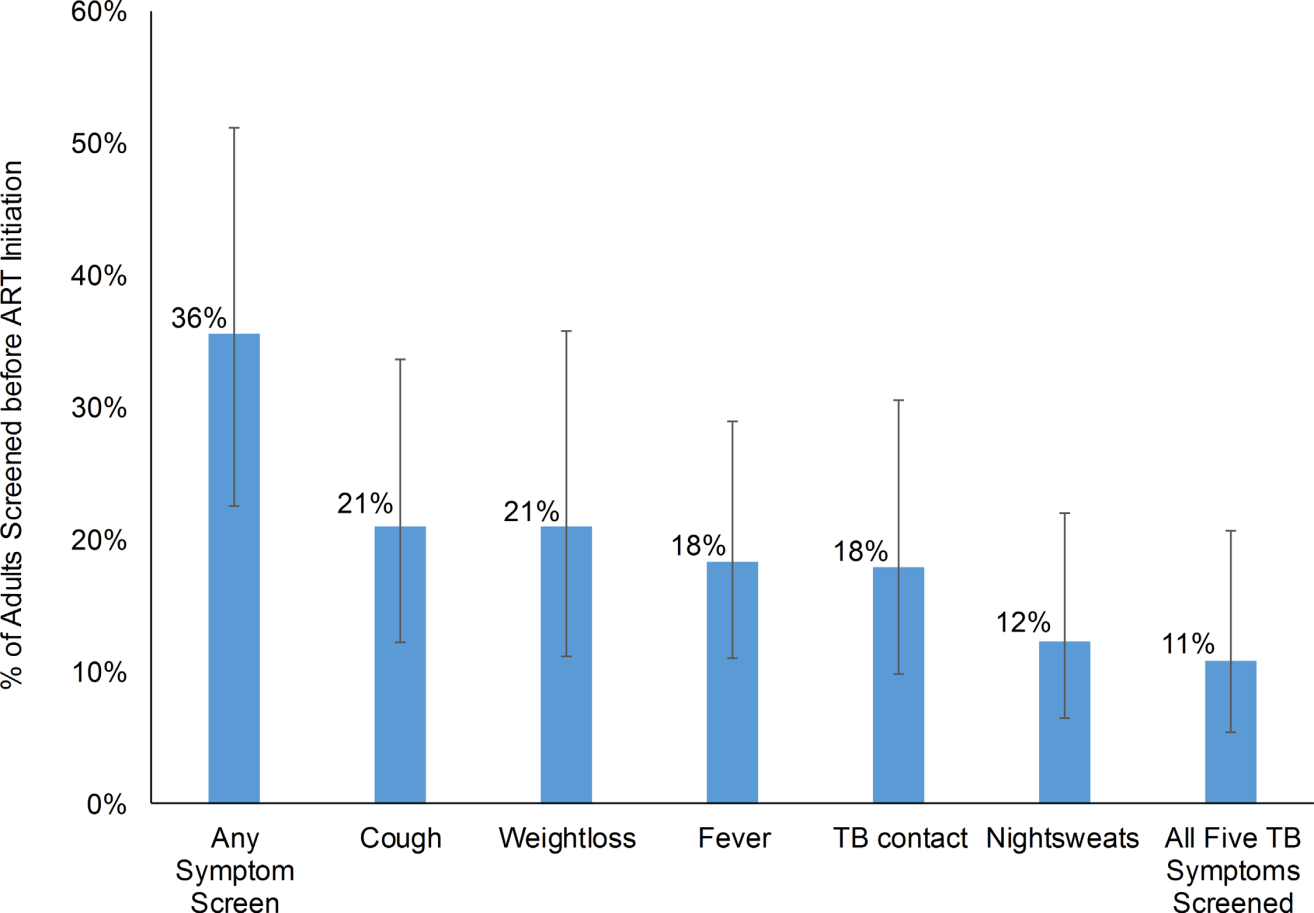
Percentages of Adults with Documentation of Screening for the Recommended Five Tuberculosis Symptoms before Antiretroviral Therapy Initiation in Côte d’Ivoire during 2004–2007*. *Error bars represent 95% confidence intervals derived using survey procedures that accounted for study design.

Among all 3,682 enrollees, 73 (2%) were on TB treatment at the time of referral to the ART facility; TB screening and diagnostic practices for these 73 patients were not documented in the ART medical records. Among the 3,609 not on TB treatment, 1,263 (36%) were documented to receive some TB screening before ART initiation at the ART facility; 21% were screened for cough, 21% for weight loss, 18% for fever, 18% for TB contacts, and 12% for night sweats (Fig 1). All five TB screening questions were only administered to 11% of patients. Documentation of who screened positive for one or more TB symptoms was not a required field in standard MOH ART charts and therefore could not be abstracted. However, of 1,263 patients screened for TB, 105 (8%) were documented to provide sputum for smear microscopy, 360 (25%) received a chest X-ray, 418 (30%) either provided a sputum sample for microscopy or received a chest X-ray, and 47 (3%) both provided a sputum sample and received a chest X-ray. Among the 1,263 with documented TB symptom screening, 111 (11%) were ultimately diagnosed with TB and started TB treatment before ART initiation. Among the 2,346 who had no documentation of TB screening before ART, zero patients started TB treatment before ART. Of the 111 TB diagnoses, 32 (25%) had provided at least one sputum sample for smear microscopy, 53 (46%) had received an X-ray, 7 (5%) had provided sputum for microscopy and received an X-ray, and 33 (35%) had no documentation of providing sputum or being X-rayed.

### Predictors of being Screened for TB

**Fig 2:**
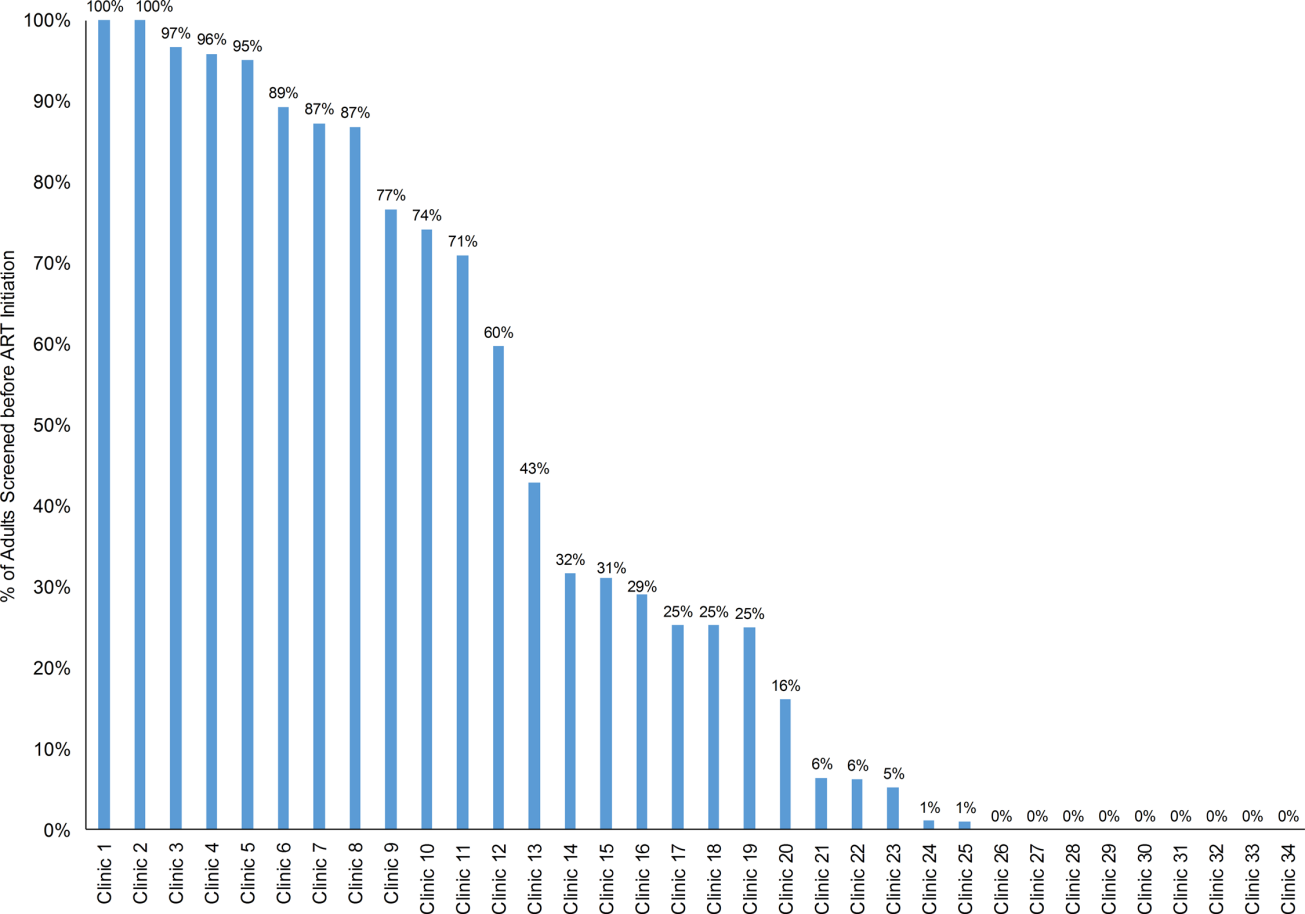
Facility-Level Variations in the Percentage of Adults Screened for Tuberculosis before Antiretroviral Therapy Initiation in Côte d’Ivoire during 2004–2007.

No associations between patient characteristics and probability of observing TB symptom screening documentation in the medical record were observed (S1 Table). No significant changes in the proportion of ART patients documented to have received any form of TB screening from 2004 to 2007 in Côte d’Ivoire were noted with 44% screened in 2004, 31% in 2005, 33% in 2006, and 38% in 2007. However, significant variation in pre-ART TB screening documentation between facilities was observed with the percentage of medical records documenting screening ranging from 0% to 100% (Fig 2); 25 (74%) of 34 facilities documented at least some pre-ART TB screening, however, only eight (24%) of 34 facilities documented pre-ART TB symptom screening for more than 80% of their ART enrollees (Fig 2).

### Predictors of Active TB at ART Initiation

A total of 184 (6%) patients (95% CI, 2–9%) were diagnosed with and treated for active TB at ART initiation (Table 1). Having more advanced WHO disease stage at ART initiation was associated with active TB at ART enrollment (p <0.001).

**Table 1:** Associations between Patient Characteristics and Prevalence of Tuberculosis Treatment at ART Initiation.

		Original				Imputed Data						
		All patients at enrollment (n = 3,682)				All patients at enrollment		No TB (n = 3,498)		TB (n = 184)		P-value
		n	N	Median/%	IQR/CI	Median/%	IQR/CI	Median/%	IQR/CI	Median/%	IQR/CI	
Age at enrollment Median (IQR) year		3,682	3,682	36	(31–43)	36	(31–43)	36	(30–43)	36	(31–43)	0.636
Sex	Female	2,422	3,682	67%	(63–70)	67%	(63–70)	67%	(63–70)	64%	(51–77)	0.601
	Male	1,260	3,682	33%	(30–37)	33%	(30–37)	33%	(30–37)	36%	(23–49)	
Marital Status												
	Civil union/married	1,636	3,268	50%	(46–55)	50%	(46–53)	49%	(46–53)	52%	(43–61)	0.514
Single/widowed/divorced		1,632	3,268	50%	(47–54)	50%	(47–54)	51%	(47–54)	48%	(39–57)	
	Missing	414	3,682	11%								
Employment												
	Employed	1,601	2,601	61%	(56–66)	59%	(54–64)	59%	(54–64)	60%	(47–73)	0.888
	Student	75	2,601	3%	(1–4)	2%	(1–4)	3%	(1–4)	1%	(0–4)	
	Unemployed	925	2,601	37%	(32–41)	39%	(34–44)	39%	(34–44)	39%	(25–52)	
	Missing	1,081	3,682	29%								
World Health Organization Stage												
	Stage I/II	587	2,581	20%	(7–44)	20%	(0–40)	21%	(1–41)	4%	(0–10)	**<0.001**
	Stage III	1,440	2,581	58%	(44–71)	58%	(44–72)	59%	(44–75)	33%	(13–53)	
	Stage IV	554	2,581	22%	(14–34)	22%	(13–31)	19%	(12–27)	63%	(40–86)	
	Missing	1,101	3,682	30%								
Weight												
	<45kg	625	3,256	20%	(17–23)	20%	(17–23)	20%	(16–23)	21%	(12–29)	0.079
	45-60kg	1,823	3,256	56%	(54–58)	56%	(53–58)	55%	(53–57)	63%	(51–76)	
	>60 kg	808	3,256	24%	(20–29)	24%	(20–29)	25%	(21–29)	16%	(8–24)	
	Missing	426	3,682	12%								
CD4 Count												
	<50 cells/μL	788	3,343	24%	(21–26)	24%	(21–26)	24%	(21–26)	25%	(16–35)	0.560
	50–<200 cells/μL	1,507	3,343	45%	(43–48)	45%	(43–48)	45%	(42–48)	46%	(39–53)	
	≥200 cells/μL	1,048	3,343	31%	(29–34)	31%	(29–34)	31%	(29–34)	29%	(21–36)	
	Missing	339	3,682	8%								
Hemoglobin												
	<8 g/dL	387	3,149	13%	(11–15)	14%	(11–16)	14%	(11–16)	12%	(6–19)	0.236
	≥8 g/dL	2,762	3,149	87%	(85–89)	86%	(84–89)	86%	(84–89)	88%	(81–94)	
	Missing	533	3,682	14%								
Co-trimoxazole												
	Prescribed	2,080	3,682	59%	(46–70)	59%	(46–71)	59%	(46–71)	56%	(38–75)	0.737
	Not prescribed CTX	1,602	3,682	41%	(30–54)	41%	(29–54)	41%	(29–54)	44%	(25–62)	
Adherence												
	≥95% adherent	887	1,413	67%	(57–76)	66%	(55–76)	65%	(55–75)	73%	(58–88)	0.217
	<95% adherent	526	1,413	33%	(24–43)	34%	(24–45)	35%	(25–45)	27%	(12–42)	
	Missing	2,269	3,682	62%								
HIV Type	HIV-1	3,464	3,646	95%	(94–96)	95%	(94–96)	95%	(94–96)	97%	(93–100)	0.580
	HIV-2	82	3,646	2%	(1–3)	2%	(1–3)	2%	(2–3)	1%	(0–2)	
	Both HIV-1&2 Reactive	100	3,646	3%	(2–4)	3%	(2–4)	3%	(2–4)	3%	(0–5)	
	Missing	36	3,682	1%								
Site Size	** **											
	>1,000 enrollees ever	2,147	3,682	51%	(29–72)	51%	(29–72)	52%	(29–75)	37%	(5–69)	0.273
** **	≤ 1,000 enrollees ever	1,535	3,682	49%	(28–71)	49%	(28–71)	48%	(25–71)	63%	(31–95)	
Any Stock out in last 12 months												
	Yes	2,307	3,682	63%	(26–89)	63%	(26–89)	62%	(25–100)	72%	(41–100)	0.45
	No	1,375	3,682	37%	(11–74)	37%	(11–74)	38%	(0–75)	28%	(0–59)	
Patient: Health Care Worker Ratio												
** **	< 100	1,499	3,682	42%	(22–65)	42%	(22–65)	41%	(17–64)	56%	(21–91)	0.278
	≥ 100	2,183	3,682	58%	(35–78)	58%	(35–78)	59%	(36–83)	44%	(9–79)	

Abbreviations: IQR, inter-quartile range; CI, confidence interval; n, numerator for weighted percentage; N, denominator for weighted percentage.

### ART Regimen Prescription Practices

ART regimen choice was affected by diagnosis of TB at ART initiation. Among HIV-1-infected patients, compared with patients not diagnosed with TB at ART initiation, a higher proportion of patients with prevalent TB were prescribed efavirenz-containing regimens (64% vs. 34%) (Table 2). Among HIV-2-infected and dually HIV-1&2-reactive patients, correct prescription of two nucleoside reverse transcriptase inhibitors (NRTI) and a protease inhibitor (PI) was more common among TB co-infected patients, compared with patients not diagnosed as having TB (61% vs. 46%).

**Table 2:** First Line Antiretroviral Therapy Regimen Distribution by Tuberculosis Treatment Status and HIV Type at Antiretroviral Therapy Initiation in Côte d’Ivoire during 2004–2007.

	n	N	%	95% CI	No TB at ART Initiation	TB at ART Initiation	P-value
**HIV-1-infected**							
D4T+3TC+NVP	1,640	3,464	51%	(37%–65%)	53%	23%	<0.001
AZT+3TC+NVP	87	3,464	3%	(1%–8%)	3%	0%	
D4T+3TC+EFV	769	3,464	26%	(18%–36%)	24%	56%	
AZT+3TC+EFV	304	3,464	10%	(6%–15%)	10%	12%	
Triple NRTI	115	3,464	3%	(1%–5%)	3%	1%	
2 NRTI's + PI	94	3,464	2%	(2%–3%)	2%	1%	
Sub-optimal Regardless of HIV Type*	125	3,464	5%	(2%–10%)	5%	6%	
Other	14	3,464	1%	(0%–2%)	0%	1%	
Missing	316	3,780	8%				
							
**HIV-2 or Dually HIV-1&2-Infected**							
D4T+3TC+NVP	28	218	9%	(3%–21%)	9%	0%	0.912
AZT+3TC+NVP	7	218	3%	(1%–10%)	3%	0%	
D4T+3TC+EFV	26	218	14%	(6%–30%)	15%	0%	
AZT+3TC+EFV	11	218	6%	(2%–15%)	6%	16%	
Triple NRTI	27	218	11%	(5%–22%)	11%	23%	
2 NRTI's + PI	99	218	46%	(37%–56%)	46%	61%	
Sub-optimal Regardless of HIV Type*	14	218	10%	(4%–22%)	10%	0%	
Other	1	218	1%	(1%–1%)	1%	0%	
Missing	5	223	2%				

Abbreviations: D4T, stavudine; 3TC, lamivudine; NVP, nevirapine; AZT, zidovudine; EFV, efavirenz; NRTI, nucleoside reverse transcriptase inhibitor; PI, protease inhibitor; CI, confidence interval; TB, tuberculosis;; n, numerator for weighted percentage; N, denominator for weighted percentage.

### Incident Tuberculosis

The study did not collect data on TB symptom screening post ART initiation due to funding and feasibility restrictions. However, during ART, 117 patients were documented to be diagnosed with and treated for TB. There were 98 cases of pulmonary TB and 19 cases of extra-pulmonary TB. The overall rate of documented incident TB was 3.0/100 person-years (PY). TB incidence was highest in the first 3 months of ART (5.5/100 PY), and declined to 2.0/100 PY, 1.2/100 PY, and 0.7/100 PY during the time periods of 3–6 months, 6–12 months, and >12 months of ART, respectively.

**Fig 3:**
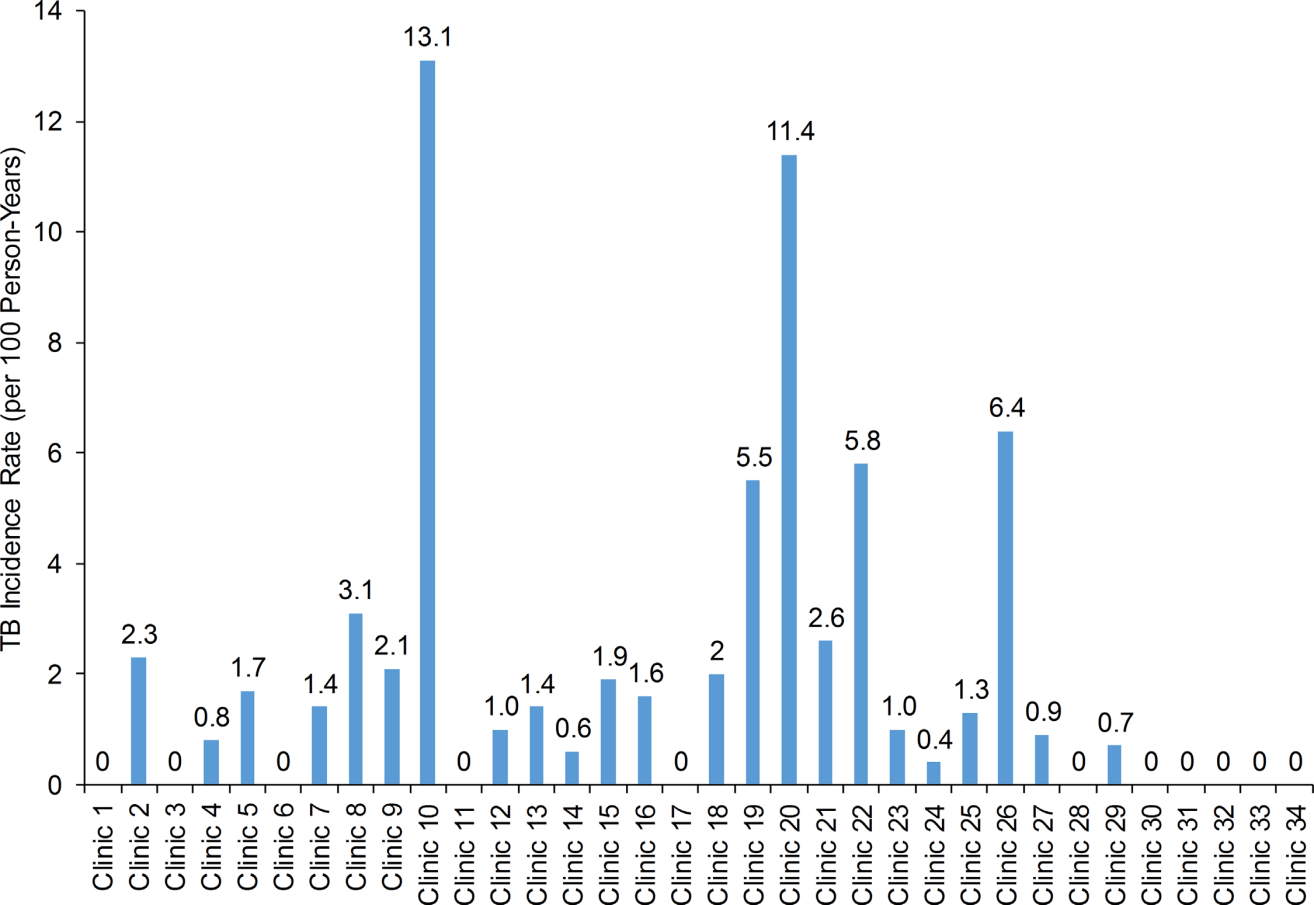
Variations in the Incidence Rate of Tuberculosis during Antiretroviral Therapy between Health Facilities in Côte d’Ivoire during 2004–2008.

Although none of the measured patient-level and site-level covariates were associated with incident TB (S2 Table), wide variations in incident TB were observed between ART facilities, with TB incidence ranging from 0 per 100 person-years to 13.1 per 100 person-years (Fig 3). Associations between available site-level characteristics and site-level TB incidence were explored, but none were statistically significant (S2 Table). No statistically significant association was observed between site-level pre-ART TB symptom screening documentation compliance and site-level TB incidence rates after ART initiation.

## Discussion

This is the first nationally representative study of TB screening documentation, prevalent TB, and TB incidence during ART in Côte d’Ivoire’s adult ART program and has several important findings.

### TB Screening and Diagnostic Practices

Documentation of TB symptom screening before ART was often missing with only 36% of medical records documenting screening for at least one TB symptom and only 11% of records documenting screening for all five symptoms recommended by national guidelines. Even though it is possible that some patients were screened for TB symptoms but screening was not documented, this still represents incomplete adherence to national TB screening policy, which requires both TB screening implementation and documentation [19,20]. Low rates of documented TB screening at ART facilities have been reported from other countries, including Mozambique, where only 61% of patients were documented to have been screened for TB before ART and only 5% were documented to have been screened for all five MOH-recommended TB symptoms [8]. TB symptom screening is important because diagnosis of TB and initiation of TB treatment before ART can reduce TB-related morbidity and mortality during ART [21], and can contribute to both nosocomial and community reductions in TB transmission [22]. In addition, exclusion of active TB can allow initiation of isoniazid preventive therapy (IPT), which has been shown in Côte d’Ivoire [9] and other countries [23] to reduce incidence of active TB and mortality. If Côte d’Ivoire is to adopt WHO recommendations for universal IPT prescription to HIV-infected persons, significant improvement in TB screening documentation is desirable so that the MOH can appropriately monitor and evaluate rollout of the IPT policy [22].

Notably, there were no patient-level predictors of screening documentation, suggesting that clinicians were not preferentially documenting TB screening based on clinical perceptions of the likelihood of having TB. Instead, variations in TB screening documentation were noted at the facility-level, similar to other studies [8]. Variations in investment in TB-screening training and monitoring programs at the site-level might therefore explain the variations in TB screening documentation observed in this study [8]. Site-level improvement activities targeting under-performing facilities, might be the most efficient way to improve overall TB screening implementation and documentation.

Per national guidelines, all patients screening positive for one or more TB symptom should have submitted a sputum sample for smear microscopy. MOH ART charts do not record who screened positive for one or more TB symptoms and this is something that could be modified to facilitate future monitoring of TB screening and diagnostic practices. However, our data show that 25% of patients screened for TB symptoms received a chest X-ray, whereas only 8% were documented to provide sputum for diagnosis. This suggests possible low compliance with sputum sample collection from presumptive TB patients or poor documentation of sputum collection, a finding which has been observed in other countries, including routine settings in South Africa [24].

Possible reasons for low documented compliance with sputum collection from presumptive TB patients include: (1) lack of smear microscopy services at many ART clinics, (2) clinician frustration with long sample turnaround times or lost samples, (3) clinician knowledge that a negative smear microscopy result for an HIV-positive presumptive TB patient carries low negative predictive value [25], (4) difficulty collecting sputum samples from presumptive TB patients who might not be coughing [24], (5) lack of a designated outdoor coughing spot or patient stigma associated with using the coughing spot [26], or (6) insufficient training in the national TB diagnostic algorithm. A consequence of low sputum collection rates is that, of the 111 new TB diagnoses prior to ART initiation, only 32 (25%) were documented to have provided sputum, suggesting that at least 75% of TB diagnoses were not bacteriologically confirmed and TB treatment was started based on clinical picture alone, or clinical picture in conjunction with X-ray findings. Further research into reasons for non-compliance with sputum collection policy is warranted as the new Xpert MTB/RIF assay is rolled out; continued failure to collect sputum samples from presumptive TB patients would significantly blunt any impact of the improved TB diagnostic [24,27].

This study observed a 6% TB prevalence overall among all 3,682 ART enrollees, with 111 TB patients diagnosed after TB screening at the ART clinic but before ART initiation, and 73 TB patients diagnosed prior to referral to the ART clinic. This overall prevalence is similar to other routine program reports from Côte d’Ivoire [11]. However, TB was diagnosed and treatment started for 111 (11%) of the 1,263 patients with some documentation of TB screening before ART. Since our data show that clinicians were not preferentially screening sicker patients, it might be reasonable to assume that 11% of the 2,346 patients who were not screened for TB before ART, might have been diagnosed and treated for TB if they had been screened for TB. This would mean that about 258 (11% x 2,346) cases of prevalent TB might have been missed before ART initiation. With a possible total of 442 TB cases at ART initiation (184 of whom were diagnosed before ART and 258 of whom remained undiagnosed at ART start), this suggests as many as 58% (258/442) of prevalent TB cases might have been missed before ART initiation. Given that TB screening and diagnostic procedures are not 100% sensitive, an even higher proportion of prevalent TB cases might have been missed. Patients with undiagnosed TB at ART initiation are at risk of Immune Reconstitution Inflammatory Syndrome (IRIS) due to unmasking of TB symptoms or worsening of existing TB symptoms, since TB symptoms are dependent on both bacillary burden and immune response [28]. In addition, undiagnosed TB is thought to be a common cause of death during ART [6,7,29]. Therefore, improvements in TB screening before ART could significantly reduce morbidity and mortality during ART, which is an important priority for ART program managers.

### Importance of Early ART

Similar to other studies [8,10], markers of advanced disease were predictive of prevalent TB at ART initiation, and TB incidence declined sharply over time after ART was started. Both these findings highlight the importance of early ART initiation; earlier diagnosis, linkage to HIV care, and early ART initiation can reduce TB morbidity for the patient [9], and contribute to reductions in TB incidence in the community [30].

### Antiretroviral Therapy Regimens

Because Rifampicin is a potent inducer of many genes controlling drug metabolism and transport, including the Cytochrome P450 iso-enzymes, and appears to reduce plasma levels of nevirapine more than efavirenz, the WHO recommends using efavirenz in initial ART regimens for patients concurrently taking TB treatment [31]. In line with these guidelines, efavirenz was more commonly prescribed to HIV-1-infected patients with prevalent TB compared with patients not on TB treatment at ART initiation (68% vs. 34%).

Among HIV-2-infected and dually HIV-1&2-infected patients, 30% of HIV-2-infected or dually HIV-1&2-reactive patients were incorrectly prescribed non-nucleoside reverse transcriptase inhibitors (NNRTIs), antiretrovirals to which HIV-2 is inherently resistant [32]. Surprisingly, co-infection with TB was associated with correct prescription of two nucleoside reverse transcriptase inhibitors (NRTIs) and a protease inhibitor (PI), with 61% of TB co-infected patients versus 46% of patients not taking TB treatment prescribed this regimen. This may indicate that TB co-infected patients living with HIV-2 or dual HIV-1&2-infection were managed at facilities with greater expertise in HIV-2 management. Similar to previous reports, these findings suggest training in regimen prescription practices for HIV-2 or dually infected patients could improve the quality of HIV-2 care [13].

### Predictors of TB Incidence

Lack of an association between patient-level risk factors for TB and incident TB may be due to insufficient sample size, or measurement error. Nine of 34 facilities reported zero TB incidence during ART which suggests that either clinicians were failing to screen, diagnose and treat TB or clinicians were failing to document incident TB cases. Among facilities that did document some TB incidence, TB incidence still varied greatly by site, ranging from 0.4/100 PY to 13.1/100 PY. The high incidence rates at some facilities are concerning. TB incidence rates of 13.1/100 PY correspond to an incidence rate of about 13,100 TB cases/100,000 population which is 79 times higher than the national average of 165/100,000. Associations between site-level characteristics and site-level TB incidence were explored, and although scatter plots suggested a possible association between lower median site-level CD4 count at ART initiation and higher TB incidence, this association was not statistically significant. Possible explanations for higher-than-expected TB incidence rates at some facilities might include lower thresholds for empiric TB treatment at some facilities, or problems with the diagnostic cascade (e.g., higher than expected false positive TB diagnoses from smear microscopy). Alternately, these facilities might truly be “hot-spots” for active TB acquisition and transmission. Sub-optimal adherence to ART might result in sub-optimal CD4 response, which in turn increases TB acquisition risk [8,30]. Alternately, poor facility-level infection control (e.g. poor ventilation of waiting areas or no designated outdoor “cough spot”) could mean that transmission of TB at the facility is increased among ART enrollees. Another explanation is that community-level TB transmission rates are higher in the community served by the facility. Further research to explore facility variations in TB incidence rates is warranted to facilitate a programmatic response.

### Limitations

This study has several limitations. Firstly, true TB incidence rates may be higher than reported here, due to missed diagnoses caused by inconsistent screening, insensitive TB diagnostic tests, poor documentation practices, or rare instances where TB may have been diagnosed but treatment not started. However, true TB incidence might also be lower since some patients might have been prescribed empiric TB treatment when they did not have active TB disease. Secondly, insufficient sample size might have resulted in insufficient power to detect true associations. Thirdly, low observed compliance with TB screening guidelines may be due, in part, to clinician failure to document their TB screening activities. Fourthly, due to funding and feasibility restrictions, this study did not evaluate documentation of TB symptom screening after ART initiation. Finally, our follow-up period ended in 2010 and country-wide rates of TB incidence and prevalence may have changed in more recent years.

### Conclusion

In this first nationally representative survey of TB screening practices in ART facilities in Côte d’Ivoire, documentation of TB screening before ART initiation was missing from most ART charts. The percentage of ART charts documenting some TB screening before ART varied from 0–100% suggesting that targeting under-performing facilities with site-improvement activities may be the most efficient approach to improving TB screening implementation and documentation, which could reduce TB-related morbidity and mortality, as well as nosocomial TB transmission. Training and mentoring in ART prescription practices, especially for HIV-2 and dually HIV-1&2-infected patients is needed. Earlier diagnosis, linkage to care, and ART initiation could reduce TB burden among patients and also contribute to reductions in nosocomial and community TB transmission. Wide variations in TB incidence by ART facility warrant further research; these variations might reflect differences in compliance with TB screening and diagnostic practices, differences in TB case documentation, or true differences in active TB risk due to differences in nosocomial or community-level TB transmission rates.

## Supporting Information

S1 Table Predictors of Being Screened for Tuberculosis before Antiretroviral Therapy Initiation in Côte d’Ivoire during 2004–2007. (PDF)

S2 Table Predictors of Incident Tuberculosis during Antiretroviral Therapy among Antiretroviral Therapy Enrollees in Côte d’Ivoire during 2004–2007. (PDF)
