# Supplementary material for: Wide Variations in Compliance with Tuberculosis Screening Guidelines and Tuberculosis Incidence between Antiretroviral Therapy Facilities — Côte d’Ivoire
Source: PLoS One. 2016 Jun 8;11(6):e0157059. doi: 10.1371/journal.pone.0157059 (PMC4898722; doi:10.1371/journal.pone.0157059)
Supplement: S1 Table — (PDF) [file pone.0157059.s001.pdf]

S1 Table. Predictors of Being Screened for Tuberculosis before Antiretroviral Therapy Initiation in Côte d'Ivoire during 2004–2007

		Not Screened for TB (N=2,346)* Median/%	Screened for TB (N=1,263)* Median/%	Unadjusted Odds ratio	95% CI	P-value
Age at enrollment						
	Median (IQR) year	36	36	1.00	(0.99-1.01)	0.712
Sex						
	Male	64%	36%	1.00	--	--
	Female	65%	35%	0.96	(0.78-1.19)	0.708
Marital Status						
	Civil union/married	62%	38%	1.00	--	--
	Single/widowed/divorced	66%	34%	0.84	(0.65-1.1)	0.198
Employment						
	Employed	66%	34%	1.00	--	--
	Student	71%	29%	0.79	(0.38-1.67)	0.521
	Unemployed	62%	38%	1.15	(0.87-1.52)	0.297
WHO Stage						
	Stage I/II	68%	32%	1.00	--	--
	Stage III	67%	33%	1.08	(0.58-2.03)	0.792
	Stage IV	55%	45%	1.78	(0.73-4.35)	0.194
Weight						
	<45kg	61%	39%	1.00	--	--
	45-60kg	64%	36%	0.87	(0.66-1.14)	0.292
	>60 kg	69%	31%	0.70	(0.46-1.07)	0.095
CD4 Count						
	<50 cells/µL	65%	35%	1.00	--	--
	50 - <200 cells/µL	65%	35%	1.02	(0.8-1.29)	0.898
	≥200 cells/µL	63%	37%	1.10	(0.89-1.37)	0.345
Hemoglobin						
	<8 g/dL	60%	40%	1.00	--	--
	≥8 g/dL	65%	35%	0.80	(0.62-1.04)	0.090
Co-trimoxazole						
	Prescribed	63%	37%	1.00	--	--
	Not prescribed CTX	66%	34%	0.86	(0.44-1.67)	0.638
Adherence						
	≥95% adherent	63%	37%	1.00	--	--
	<95% adherent	68%	32%	0.80	(0.35-1.83)	0.578
HIV Type						
	HIV-1	64%	36%	1.00	--	--
	HIV-2	66%	34%	0.93	(0.62-1.4)	0.714
	Both HIV-1&2 reactive	62%	38%	1.10	(0.64-1.89)	0.710
Site Size						
	>1,000	65%	35%	1.00	--	--
	≤ 1,000	64%	36%	1.03	(0.39-2.71)	0.952
Any Stock out						
	No	60%	40%	1.00	--	--
	Yes	72%	28%	0.57	(0.21-1.56)	0.260
Patients:HCW						
	P:HCW ≥ 100	65%	35%	1.00	--	--
	P:HCW < 100	64%	36%	1.05	(0.37-2.96)	0.931
Nurse Satisfaction						
	Generally satisfied	62%	38%	1.00	--	--
	Dissatisfied	67%	33%	0.81	(0.29-2.29)	0.684
Doctor Satisfaction						
	Generally satisfied	61%	39%	1.00	--	--
	Dissatisfied	68%	32%	0.74	(0.26-2.06)	0.543

Abbreviations: TB, tuberculosis; OR, odds ratio; CI, confidence interval; IQR interquartile range; WHO, world health organization; CTX, co-trimoxazole; HCW, health care worker; P:HCW, patient:health care worker ratio.

*Analysis excluded the 73 patients who were referred to the ART clinic after having started TB treatment, and for whom TB screening would not have been necessary.
