# Supplementary material for: Wide Variations in Compliance with Tuberculosis Screening Guidelines and Tuberculosis Incidence between Antiretroviral Therapy Facilities — Côte d’Ivoire
Source: PLoS One. 2016 Jun 8;11(6):e0157059. doi: 10.1371/journal.pone.0157059 (PMC4898722; doi:10.1371/journal.pone.0157059)
Supplement: S2 Table — (PDF) [file pone.0157059.s002.pdf]

S2 Table. Predictors of Incident Tuberculosis during Antiretroviral Therapy among Antiretroviral Therapy Enrollees in Côte d'Ivoire during 2004–2007

	Original		Crude			Adjusted		
	No	Rate/100	HR	(95% CI)	P	AHR	(95% CI)	P
Age at enrollment								
Per 10-year increase	3,682		0.96	(0.66-1.41)	0.846	0.98	(0.64-1.5)	0.926
Sex								
Female	2,422	1.38	1.00	––	––	1.00	––	––
Male	1,260	1.49	1.05	(0.72-1.53)	0.805	1.22	(0.71-2.1)	0.464
Marital Status								
Civil union/married	1,636	1.33	1.00	––	––	1.00	––	––
Single/widowed/divorced	1,632	1.49	1.11	(0.77-1.59)	0.557	1.11	(0.73-1.68)	0.606
Employment								
Employed	1,601	1.31	1.00	––	––	1.00	––	––
Student	75	2.11	1.55	(0.44-5.48)	0.475	1.47	(0.39-5.45)	0.548
Unemployed	925	1.53	1.13	(0.67-1.93)	0.624	1.17	(0.67-2.06)	0.56
WHO Stage								
Stage I/II	587	0.96	1.00	––	––	1.00	––	––
Stage III	1,440	1.47	1.49	(0.58-3.84)	0.388	1.52	(0.54-4.29)	0.412
Stage IV	554	1.72	1.67	(0.61-4.6)	0.302	1.72	(0.51-5.8)	0.363
Weight								
>60kg	808	1.30	1.00	––	––	1.00	––	––
45-60kg	1,823	1.39	1.03	(0.66-1.61)	0.882	0.93	(0.55-1.56)	0.759
<45kg	625	1.68	1.18	(0.61-2.27)	0.608	0.95	(0.38-2.34)	0.904
CD4 Count								
≥200 cells/µL	1,048	1.58	1.00	––	––	1.00	––	––
50 - <200 cells/µL	1,507	1.12	0.71	(0.44-1.15)	0.159	0.68	(0.41-1.13)	0.127
<50 cells/µL	788	1.81	1.11	(0.71-1.74)	0.637	1.02	(0.63-1.63)	0.944
Hemoglobin								
≥8 g/dL	2,762	1.33	1.00	––	––	1.00	––	––
<8 g/dL	387	2.01	1.48	(0.73-3)	0.263	1.42	(0.67-3.01)	0.347
Co-trimoxazole (CTX)								
Not Prescribed CTX	1,602	1.45	1.00	––	––	1.00	––	––
Prescribed CTX	2,080	1.39	0.96	(0.64-1.45)	0.857	0.88	(0.62-1.24)	0.455
Adherence								
≥95% adherent	887	1.29	1.00	––	––	1.00	––	––
<95% adherent	526	1.73	1.23	(0.66-2.29)	0.498	1.28	(0.7-2.32)	0.393
HIV Type								
HIV-1	3,464	1.45	1.00	––	––	1.00	––	––
HIV-2	82	0.29	0.20	(0.02-2.22)	0.178	0.21	(0.02-2.77)	0.222
Both	100	0.97	0.69	(0.2-2.36)	0.541	0.71	(0.2-2.55)	0.582
Any screening								
Yes	1,263	1.64	1.00	––	––	1.00	––	––
No	2,419	1.29	0.79	(0.42-1.49)	0.446	0.79	(0.43-1.45)	0.433
Site Size								
>1,000	2,147	1.38	1.00	––	––	1.00	––	––
≤ 1,000	1,535	1.45	0.92	(0.36-2.33)	0.847	0.93	(0.35-2.47)	0.882
Any Stock out								
No	1,375	1.35	1.00	––	––	1.00	––	––
Yes	2,307	1.44	1.09	(0.43-2.81)	0.846	1.02	(0.41-2.55)	0.97
Patients:HCW ratio								
≥100	2,183	1.41	1.00	––	––	1.00	––	––
< 100	1,499	1.41	0.86	(0.35-2.1)	0.734	0.80	(0.37-1.76)	0.569

Abbreviations: HR, hazards ratio; AHR, adjusted hazards ratio; CI, confidence interval; CTX, co-trimoxazole; WHO, World Health Organization; HCW, Health Care Worker; HR, hazard ratio; AHR, adjusted hazard ratio; CI, confidence interval
